# Global transcriptome analysis identifies differentially expressed genes related to lipid metabolism in Wagyu and Holstein cattle

**DOI:** 10.1038/s41598-017-05702-5

**Published:** 2017-07-13

**Authors:** Wanlong Huang, Yuntao Guo, Weihua Du, Xiuxiu Zhang, Ai Li, Xiangyang Miao

**Affiliations:** Mineral Nutrition Research Division, Institute of Animal Sciences, Chinese Academy of Agricultural Sciences, Beijing, 100193 China

## Abstract

Fat deposition of beef cattle varies between breeds. However, the regulation mechanism is still not elucidated completely at molecular level. In the present study, we comparatively analyzed transcriptome of subcutaneous adipose tissue between Wagyu and Holstein cattle with a significant difference in fat deposition to identify key genes associated with fat metabolism and adipogenesis by high-throughput RNA-seq technology. A total of 59,149,852 and 69,947,982 high quality reads were generated, respectively. With further analysis, 662 differentially expressed genes were identified. Gene Ontology and KEGG pathway analysis revealed that many differentially expressed genes were enriched in several biological processes and pathways relevant to adipogenesis and lipid metabolism, in which PPAR and fatty acid metabolism signaling pathways with related genes such as PPARγ, PLIN2 and ELOVL6 *et al*. play a critical role. Protein-protein interaction network analysis showed EGR1, FOS, SERPINE1, AGT, MMP2 may have great impact on adipocyte differentiation and adipogenesis. Moreover, potential alternative splicing events and single nucleotide polymorphisms (SNPs) were also identified. In summary, we comprehensively analyzed and discussed the transcriptome of subcutaneous adipose tissue of Wagyu and Holstein cattle, which might provide a theoretical basis for better understanding molecular mechanism of fat metabolism and deposition in beef cattle.

## Introduction

Meat is an important source of protein in human diet, and fat, especially intramuscular fat, plays a crucial role in meat quality including appearance, flavor, water-holding capacity, tenderness^1, 2^. However, excessive fat deposition in adipose tissue can result in obesity and energy metabolism abnormalities, which further results in obesity-associated diseases, such as Type 2 diabetes, insulin resistance, cardiovascular diseases, some cancers and so on^3^. Thus, in-depth study of molecular mechanism of fat deposition is important for breeding livestock with meat of high quality, preventing and treating diseases associated with fat metabolism.

Fat deposition of beef cattle varies between breeds. Wagyu cattle is one of the beef cattle breeds that has the highest economic and research values. The intramuscular fat deposited in its carcass is remarkable, giving its meat an appearance similar to a marble pattern^4–6^, which is widely known among people. Holstein cattle is a breed of dual-purpose (meat and milk) cattle and can also deposit intramuscular fat in its body. There are many researches on difference in fat deposition between Wagyu and Holstein cattle, which reveal that Wagyu cattle has higher percentages of subcutaneous and intramuscular fat and bigger subcutaneous and intramuscular adipose tissue cells than Holstein cattle, and differentiation activity of preadipocytes in Wagyu is also higher than that in Holstein cattle^4, 6^. However, the regulation mechanism of relatively high fat deposition and marbled meat in Wagyu cattle is still not elucidated completely at molecular level. Thus, the present study aims to investigate the molecular mechanism of difference in fat deposition between Wagyu and Holstein cattle, and further provide theoretical basis for improving beef quality and controlling obesity.

Nowadays, next-generation sequencing (NGS) has become more and more applicable. Compared with microarray chip, RNA-seq can characterize and quantitatively analyze transcriptome more comprehensively. In addition, RNA-seq has higher sensitivity in identifying differentially expressed genes and thus is the most effective method for serial analysis of gene expression^7–13^. Recently, RNA-seq has been used in whole-genome analysis of adipose tissue of sheep^14–16^, pig^17^, and cattle^18^ at transcription level, and many genes associated with fat metabolism has been identified. However, high-throughput RNA-seq has been little used to analyze adipose tissue transcriptome of Wagyu cattle, and current study is mainly focused on specific genes regulating fat deposition. Therefore, in the present study, we used RNA-seq technology to comprehensively analyze and compare the gene expression profiles of subcutaneous adipose tissue between Wagyu and Holstein cattle at transcription level, and identified many differentially expressed genes. Furthermore, with the help of Gene Ontology (GO), KEGG pathway and protein-protein interaction network analysis, we investigated the regulatory effects of differentially expressed genes on fat deposition.

## Results

### Summary of RNA-seq data

**Figure 1:**
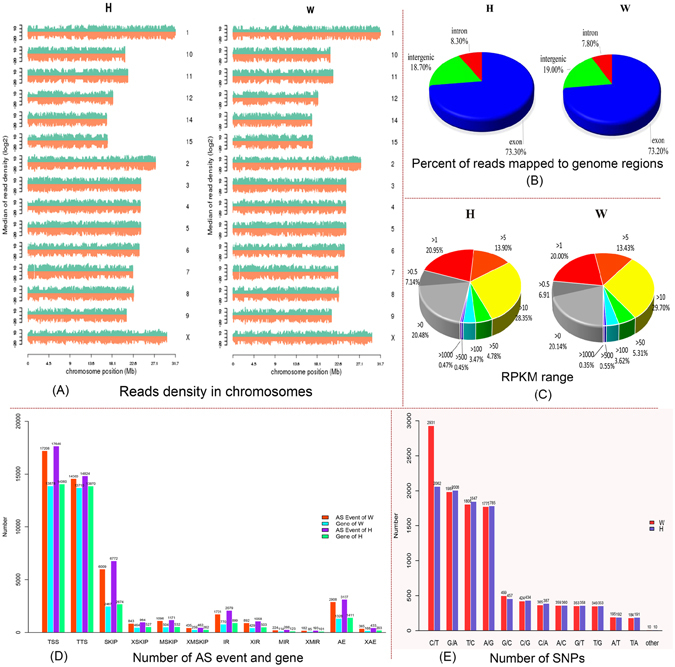
Summary of basic sequencing data. (**A**) Density of total mapped reads (W and H). X-axis represents position on chromosome (million base pairs), and Y-axis represents log2(median reads density). Green and red regions represent positive and negative strands of chromosome, respectively. (**B**) Percentage of reads mapped to different genome regions (W and H). (**C**) Percentage of genes with different expression levels (W and H). (**D**) Distribution of alternative splicing events and associated genes in W and H samples. The orange and the purple bars indicate the number of alternative splicing events identified in W and H libraries, respectively. The cyan and the spring green bars indicate the number of genes associated with each type of the 12 alternative splicing events. (**E**) Distribution of SNPs identified in W and H libraries. The red and the slate blue bars indicate the number of SNPs in W and H libraries, respectively.

By RNA-seq, 76,768,158 and 69,205,368 raw reads were obtained for Holstein (H) and Wagyu (W) libraries, respectively. After quality control of the raw reads, 69,947,982 and 59,149,852 clean reads were obtained, respectively. In H and W libraries, 62,307,204 (89.08%) and 53,130,187 (89.82%) of the clean reads were uniquely mapped to bovine reference genome Bos_taurus.UMD3.1 (ftp://ftp.ensembl.org/pub/release84/fasta/Bos_taurus/dna/) respectively (Table 1). Total mapped reads were separately were 90.19% (H) and 90.78% (W). The density of reads mapped to reference chromosome (positive and negative strands) is consistent with the length of reference chromosome (Fig. 1A), and the proportions of reads in H and W libraries mapped to exons of the reference genome are greater than 73% (Fig, 1B).

**Table 1:** Reads mapping summary.

Alignment statistics	H		W	
	counts	% of total reads	counts	% of total reads
Total clean reads	69947982		59149852	
Total mapped	63084294	90.19%	53694650	90.78%
Uniquely mapped	62307204	89.08%	53130187	89.82%
Multiple mapped	777090	1.11%	564463	0.95%
Read-1	31153457	44.54%	26243793	44.37%
Read-2	31153747	44.54%	26886394	45.45%
Reads map to ‘ + ’	31150801	44.53%	26575062	44.93%
Reads map to ‘−’	31156403	44.54%	26555125	44.89%
Non-splice reads	39936533	57.09%	34258982	57.92%
Splice reads	22370671	31.98%	18871205	31.90%

### Expression level of genes and identification of differentially expressed genes

A total of 18806 and 19329 transcripts were identified, including 1382 and 1440 newly predicated transcripts in W and H libraries, respectively. The expression levels of transcripts were normalized as reads per kilo bases per million mapped reads (RPKM) (Fig. 1C). A considerable amount of the identified genes of H and W are not expressed or have low expression levels (RPKM < 1). The majority of the identified genes have expression levels of RPKM 1–100, whereas only a few identified genes have high expression levels (RPKM > 500). We identified 662 differentially expressed genes (including 42 novel genes), which include 321 up-regulated genes and 341 down-regulated genes. There are 236 genes with 4-fold change in expression levels between W and H, which accounts for 35.6% of the differentially expressed genes (Table S1). The highly significant difference in genes are useful for studying the molecular mechanism of difference in fat deposition between Wagyu and Holstein cattle.

### Gene ontology analysis of differentially expressed genes

**Figure 2:**
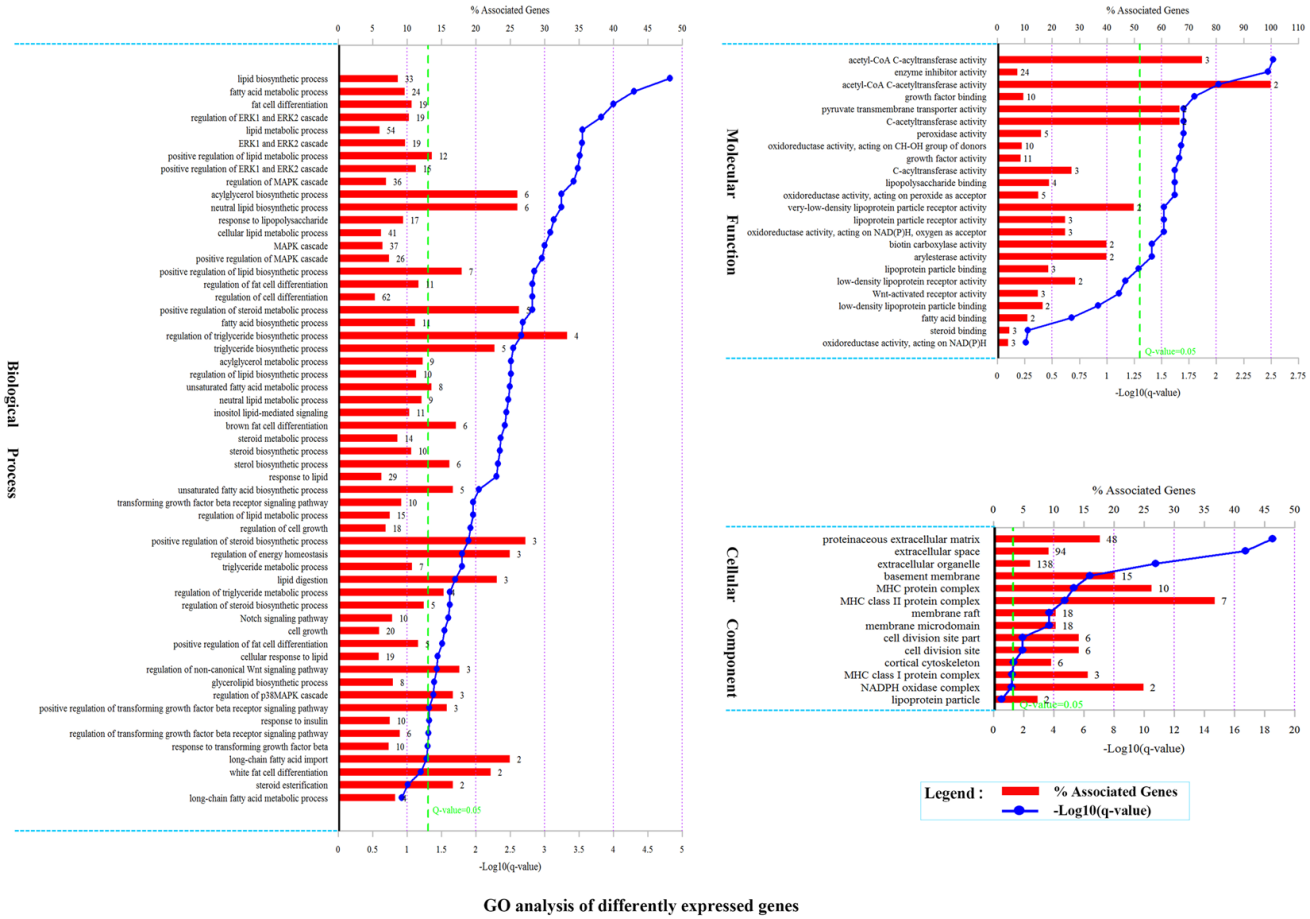
GO analysis of differentially expressed genes. The figure is composed of three parts: biological processes, molecular functions, and cellular components. The significance level of enrichment was set at corrected p_value (q_value) < 0.05.

GO enrichment analysis of differentially expressed genes was performed. Results show that 392 GO terms (15.6%, 392/2515) were significantly enriched in the biological process category, 18 GO terms (6.7%, 18/268) were significantly enriched in the molecular function category, 68 GO terms (38.0%, 68/179) were enriched in the cellular component category (Table S2). Most importantly, many of the significantly enriched GO terms are closely associated with fat metabolism and deposition. As shown in Fig. 2, in the biological process category, many genes (>20) are enriched in lipid biosynthetic process (GO:0008610), fatty acid metabolic process (GO:0006631), lipid metabolic process (GO:0006629), regulation of MAPK cascade (GO:0043408), cellular lipid metabolic process (GO:0044255), MAPK cascade (GO:0000165), positive regulation of MAPK cascade (GO:0043410), and response to lipid (GO:0033993). In addition, some of the GO terms are extremely significant enrichment (q_value < 0.001). From the molecular function prespective, many genes were enriched in acyltransferase, lipoprotein receptor activation and redox reactions. These GO terms indicate that differentially expressed genes might play important roles in fat metabolism and deposition.

### Pathway analysis of differentially expressed genes

**Figure 3:**
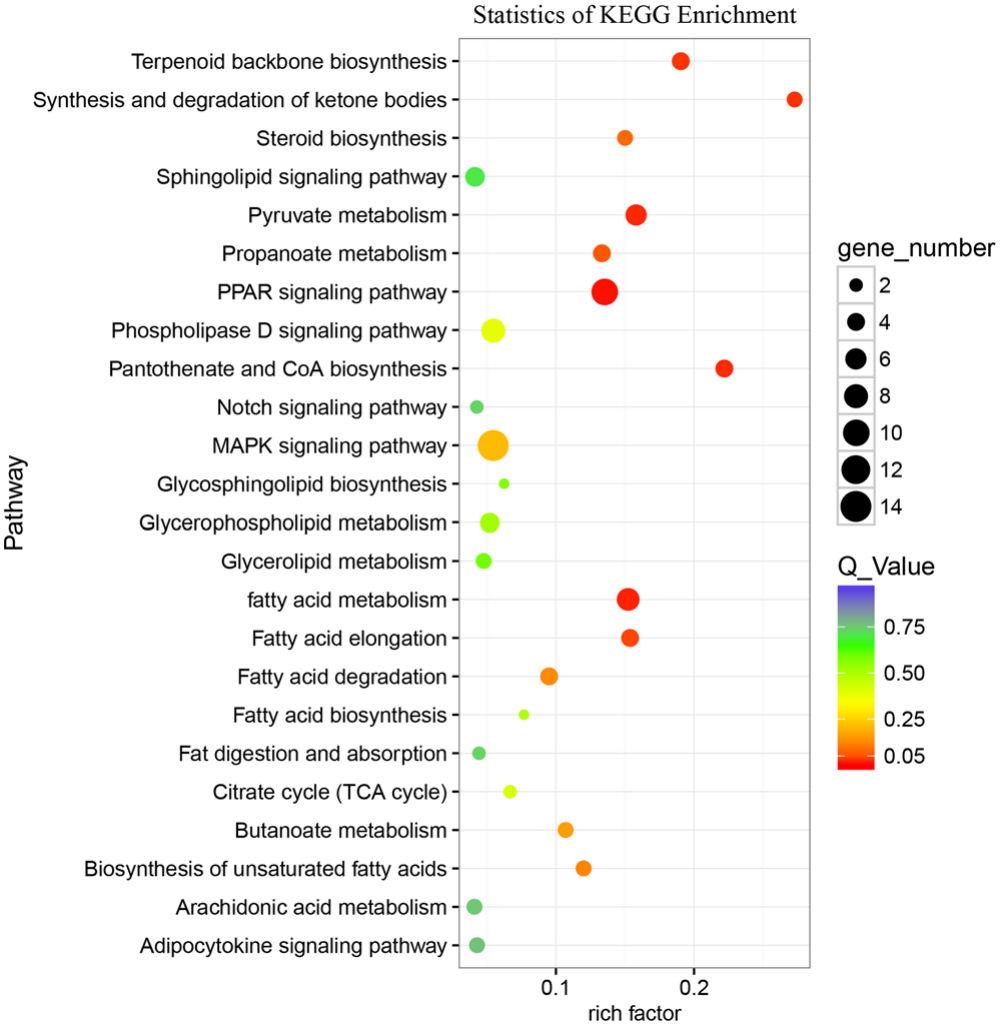
KEGG pathway analysis of differentially expressed genes. Advanced bubble chart shows enrichment of differentially expressed genes in signaling pathways. Y-axis label represents pathway, and X-axis label represents rich factor (rich factor = amount of differentially expressed genes enriched in the pathway/amount of all genes in background gene set). Size and color of the bubble represent amount of differentially expressed genes enriched in pathway and enrichment significance, respectively.

There are 154 genes significantly enriched in 47 signaling pathways (Table S3). Figure 3 shows the result of pathway enrichment associated with fat metabolism and deposition, in which PPAR signaling pathway, fatty acid metabolism, pyruvate metabolism, pantothenate and CoA biosynthesis, synthesis and degradation of ketone bodies, terpenoid backbone biosynthesis, and fatty acid elongation are significantly enriched (q_value < 0.05).

### Identification of alternative splicing events

Alternative splicing can increase transcriptome and proteome diversity, further perform complex functions and regulate gene expression^19^. Thus, alternative splicing plays an important role in many biological processes. A total of 45456 and 49004 alternative splicing events are identified in W and H libraries, respectively (Table S4). Figure 1D shows distribution of different types of alternative splicing events and genes associated with them in reference annotation file, which indicates the complexity of transcriptome in cattle. In addition, there is a consistency between amount of alternative splicing events and amount of their associated genes, and each type of alternative splicing events in H library is more than those in W. Among 12 alternative splicing events, TSS and TTS are the most common, accounting for 68.4% (31771) and 66.3% (32470) of the total alternative splicing events, followed by exon skipping (including SKIP, XSKIP, MSKIP and XMSKIP) accounting for 18.0% (8383) and 19.1% (9368), respectively.

### SNPs identification

A total of 10,289 and 10,436 potential SNPs are identified in W and H libraries, respectively. Most of the SNPs are mapped onto 30 chromosomes of cattle (29 autosomes and 1 sex chromosome) (Table S5). Among them, C/T is the most common, followed by G/A, T/C, and A/G (Fig. 1E). However, W and H libraries still have different types of SNPs.

### Protein-protein interaction network of differentially expressed genes

**Figure 4:**
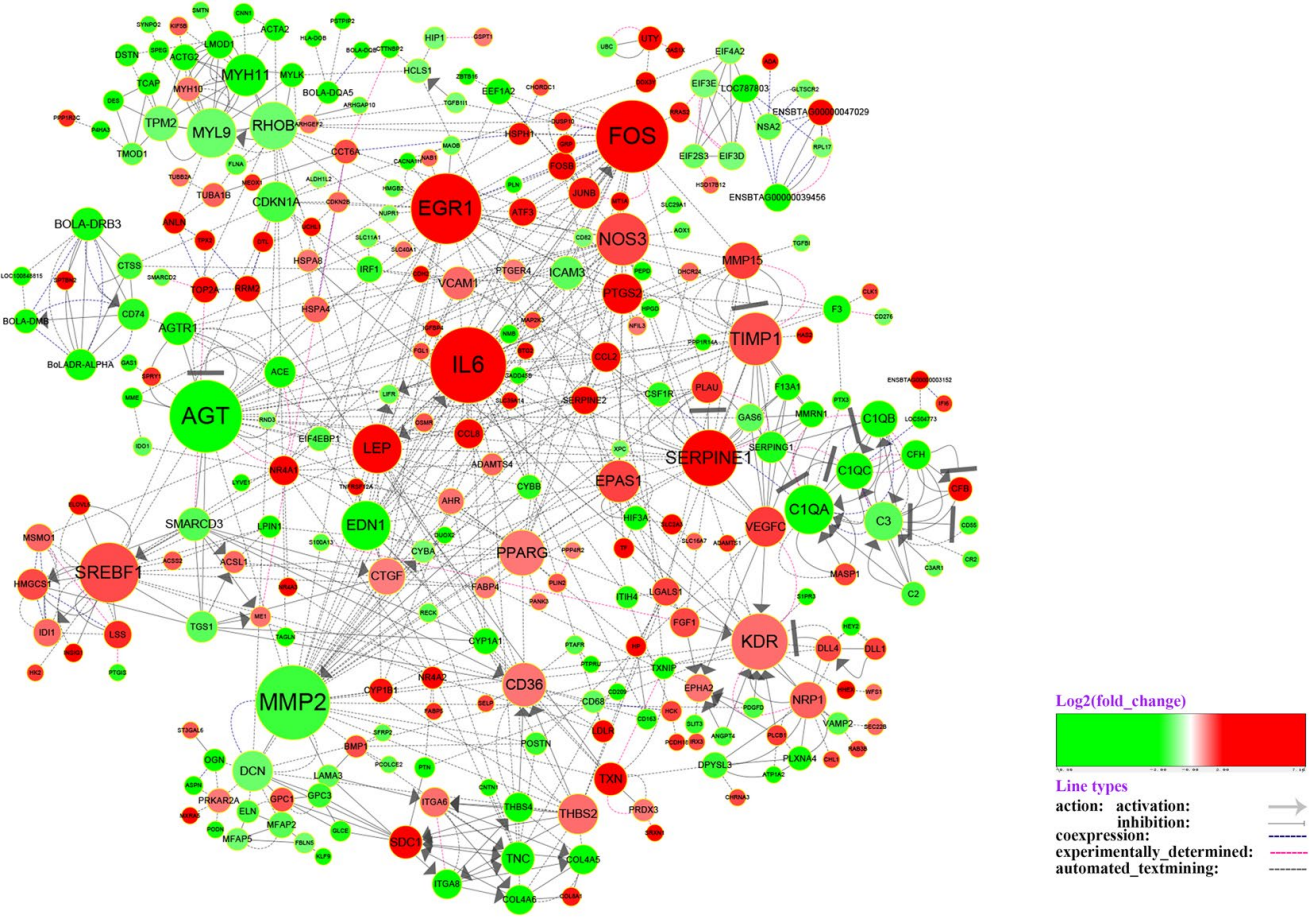
Protein-protein interaction network of differentially expressed genes. Node represents protein, edge represents interaction between proteins, size of the node is proportional to degree of this node (degree of the node is defined as amount of proteins that interact with this node), and color of node represents Log2FoldChange in expression levels of differentially expressed genes between Wagyu and Holstein cattle.

We integrated expression level of differentially expressed genes with protein-protein interaction network, and then identified the candidate key genes that potentially regulate peculiar fat deposition of Wagyu cattle, which is different from those of other cattle breeds^20^. On the basis of STRING database, interaction with score >0.6 were chosen for analysis (Table S6). As shown in Fig. 4, peroxisome proliferator activated receptor gamma (PPARγ) and fatty acid binding protein 4 (FABP4) that play crucial roles in biosynthesis of fat and fatty acid exist in interaction network. PPARγ are associated with so many genes that it is at the core position of the interaction network. In addition, some genes with significant variation are also identified, such as up-regulated genes early growth response 1 (EGR1), AP-1 transcription factor subunit (FOS), and serpin family E member 1 (SERPINE1) and down-regulated genes angiotensinogen (AGT), matrix metallopeptidase 2 (MMP2). These genes are at key positions of the interaction network.

### Validation of sequencing data by qRT-PCR

**Figure 5:**
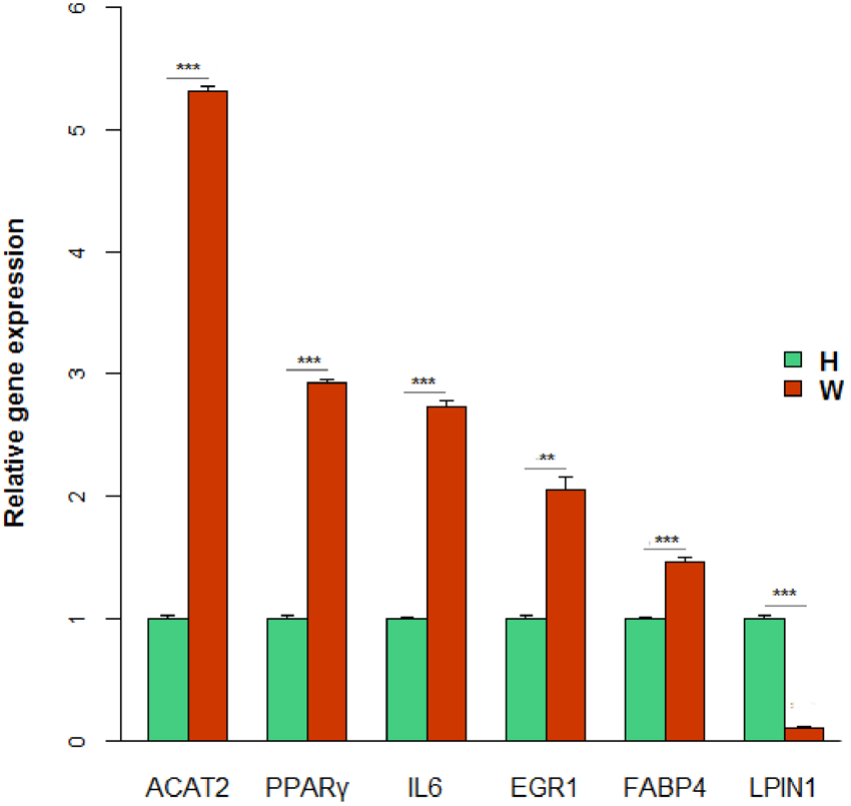
QRT-PCR validation of the differentially expressed genes. The differential expression of genes in subcutaneous fat between Wagyu and Holstein cattle was validated by quantitative real-time PCR. *P < 0.05; **P < 0.01; ***P < 0.001.

To validate the reliability of results from RNA-seq, six differentially expressed genes were randomly selected to further examined using qRT-PCR (Table S7). Figure 5 showed the result that acetyl-CoA acetyltransferase 2 (ACAT2), PPARγ, interleukin 6 (IL6), EGR1, FABP4 were significantly high expression and lipin 1(LPIN1) was significantly low expression in Wagyu cattle samples compared to Holstein. These data were in accordance with the sequencing results.

## Discussion

A lot of intramuscular fat is deposited in Wagyu carcass. Adipose tissue, especially intramuscular fat, is the key factor influencing meat quality. Some studies compared Wagyu cattle with other cattle breeds in fatty acid composition, adipocyte traits, and fat deposition^4, 6, 21–24^. On the basis of these work, we selected Wagyu and Holstein cattle for analyzing molecular mechanism of fat deposition in cattle. Recently, some researchers comparatively analyzed the expression of different adipose tissue genes of Simmental-Luxi hybrid cattle by RNA-seq, and identified many differentially expressed genes associated with fat deposition and metabolism^18^. In light of their work, we compared the expression profile of genes in subcutaneous adipose tissue between Wagyu and Holstein cattle by RNA-seq technology and comprehensively investigated fat deposition and metabolism in these cattle.

The rapid development of high-throughput sequencing technology enhances our understanding of expression profile of whole genome at transcription level. Using RNA-seq technology, we can obtain more valuable information including alternative splicing events and SNPs. In the present study, we identified many alternative splicing events and potential SNPs in W and H libraries for subcutaneous adipose tissue. Some studies also identified alternative splicing events in adipose tissues of cattle, indicating some of them could regulate adipocyte proliferation and differentiation, and promote fat biosynthesis, metabolism, and deposition^18, 25^. SNPs of genes associated with fat metabolism have great impact on carcass traits, fatty acid composition, and intramuscular fat deposition in beef cattle^26–29^. These facts imply that alternative splicing events and SNPs identified in the current study might play important roles in fat metabolism and deposition in Wagyu cattle, which needs further investigation in the future. Analysis of alternative splicing events and SNPs can help us better understand regulation mechanism of transcription in Wagyu cattle.

Differentially expressed genes are significantly enriched in signaling pathways that regulate fat metabolism and deposition. These genes involved in fatty acid metabolism pathway including acetyl-CoA acetyltransferase 2 (ACAT2), acetyl-CoA acyltransferase 2 (ACAA2), acyl-CoA synthetase long-chain family member 1 (ACSL1), acetyl-CoA acetyltransferase 1(ACAT1), ELOVL fatty acid elongase 5 (ELOVL5), ELOVL fatty acid elongase 6 (ELOVL6), hydroxysteroid 17-beta dehydrogenase 12 (HSD17B12) were up-regulated in dorsal adipose tissue of Wagyu cattle. ELOVL5 and ELOVL6 are key enzymes controlling the synthesis of long chain fatty acid, mainly promoting monounsaturated fatty acid synthesis^30^. HSD17B12 is involved in the synthesis of arachidonic acid^31^. Previous studies showed that the sum of monounsaturated fatty acid is greater and the sum of saturated fatty acid is relatively lower mainly in intramuscular fat of Wagyu compared to Holstein steers and the marbling flecks are positive correlation with the monounsaturated fatty acid content in Wagyu cattle^6, 21^. Besides, compared with other breeds, Wagyu cattle possess more unsaturated fat in subcutaneous and intramuscular depots, and reduced carcass fat can decrease the proportion of monounsaturated acid in subcutaneous fat^32^. In according with those researches, these genes significantly enriched in fatty acid metabolism in the present study may have important roles in fat biosynthesis and metabolism through regulating synthesis and elongation of the fatty acid, which also implies that the fatty acid profile of subcutaneous fat between Wagyu and Holstein cattle may be different, and dorsal adipose tissue of Wagyu cattle may process more unsaturated fatty acid.

**Figure 6:**
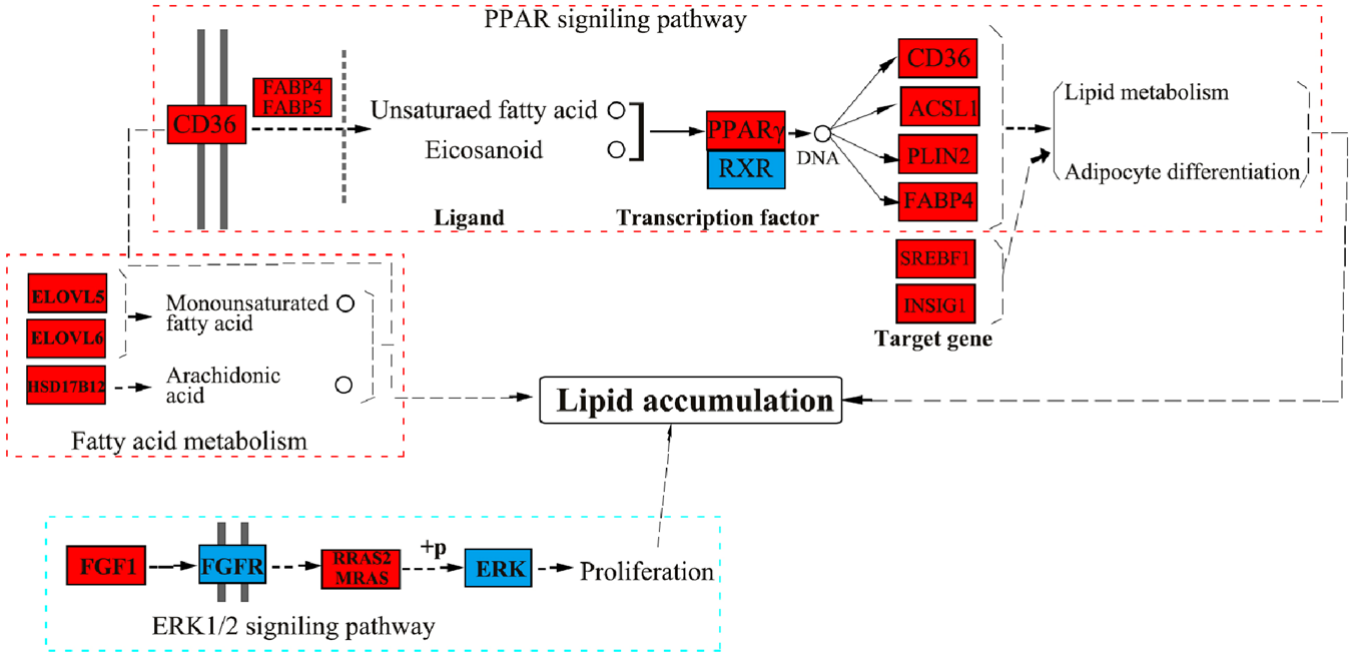
The regulatory network of lipid accumulation with significant characteristics in Wagyu cattle. The regulatory network comprised PPAR signaling pathway, fatty acid metabolism and MAPK signaling pathway in which PPAR signaling pathway and fatty acid metabolism were significant enrichment. The red oblongs represent the up-regulated genes in subcutaneous fat of Wagyu cattle. The blue oblongs represent the undifferentiated genes. SREBF1 and INSIG1 were reported as the target genes of PPARγ in previous researches. The critical part of those pathways and key genes involved were showed in the regulatory network. Those pathways drawn is mainly referred to the KEGG database (www.kegg.jp/kegg/pathway.html)^71–73^.

PPAR signaling pathway is a key pathway closely associated with metabolism of fatty acid and sterols, and adipogenic differentiation. In the current study, the core transcription factor PPARγ in PPAR signaling pathway was identified and highly expressed in the subcutaneous fat of Wagyu cattle. Previous researches have showed that PPARγ is mainly expressed in cattle adipose tissue^33^, is a key transcription factor for fat biosynthesis, and promotes intramuscular fat deposition in cattle^20^. Long chain fatty acids that function as endogenous ligands of PPARγ, can directly bind and activate PPARγ, especially unsaturated fatty acid^34, 35^. And then, activated PPARγ forms a heterodimer with retinoid X receptor alpha (RXRα), and bind to the peroxisome proliferator responsive element (PPRE) located upstream of target genes that are associated with fat metabolism and adipogenic differentiation, which regulates genes expression^36^. Therefore, more long chain fatty acids in dorsal adipose tissue of Wagyu cattle may have an important effect on the signal transduction of PPAR pathway. In the present study, five target genes of PPARγ were enriched in PPAR signaling pathway and all up-regulated in subcutaneous fat of Wagyu cattle, including FABP4, CD36 molecule/fatty acid translocase (CD36/FAT), glycerol kinase (GK), perilipin 2 (PLIN2) and ACSL1. PPARγ induces expression of FABP4 and CD36 genes. FABP4 is important for lipid hydrolysis and transportation of intracellular free fatty acids, and is closely associated with dorsal fat thickness and marbling pattern of meat in beef cattle^27^. CD36 that positively correlates with expression of PPARγ can facilitate long-chain fatty acid cellular uptake and promote adipocyte differentiation and fat biosynthesis^37^. ACSL1 act as a type of long chain fatty acid CoA ligase, which have an important role in synthesis of fatty acid and is positively correlated with lipid accumulation. PLIN2 is an important lipid droplet protein, which can enclose the surface of lipid droplet in mammalian cells and thus prevent lipase from entering lipid droplet. It can be seen that PLIN2 plays an important role in fat deposition and maintenance. In the present study, PLIN2 mRNA was highly expressed in Wagyu subcutaneous fat, which was consistent with these results, that PLIN2 mRNA was up-regulated in Hereford × Aberdeen Angus cattle (fat-type)^38^, and pig with thicker backfat^39^, which indicates PLIN2 expression may be positively correlated with backfat thickness various livestock. Besides, there is a high level of PLIN2 mRNA in intramuscular fat of Wagyu cattle compared with Holstein cattle^21^. In accordance with these results, PLIN2 may have an important role in promoting fat deposition of Wagyu cattle. It is reported that PPARγ can also target and regulate insulin-induced gene 1 (INSIG1) and sterol regulatory element binding transcription factor 1(SREBF1)^40, 41^. INSIG1 can promote biosynthesis of intramuscular fat in cattle^41^. SREBF1 plays a key role in metabolism of fatty acid, cholesterol, and lipid, and can promote adipocyte differentiation and fat synthesis by regulating enzymes associated with these processes^42–44^. Taken together, all of those genes above that highly expressed in Wagyu cattle are mainly involved in the positive regulation of adipocyte differentiation and lipogenesis, which indicates that these genes may promote subcutaneous fat deposition of Wagyu cattle, and PPAR signaling pathway may play a critical role in bovine adipogenesis in accordance with our previous study^45^. Moreover, more unsaturated fatty acid may be transported into adipocyte with the regulation of CD36, FABP4 and FABP5, then activate PPARγ and downstream genes, which finally promote adipogenic differentiation, lipid accumulation and metabolism of Wagyu cattle. Previous studies indicated that preadipocytes and stromal vascular cells derived from subcutaneous fat of Wagyu possess a higher capability of proliferation than Angus cattle^22, 24^, and phosphorylation of ERK1/2 is associated with proliferation^24^. Besides, the proliferation and differentiation capability of preadipocytes is stronger in Bamei (fat-type) than Landrace (lean-type) pig^46^. ERK1/2 belongs to MAPK signaling pathway that plays a key role in cell proliferation and differentiation. In the present study, 9 of 14 genes enriched in MAPK signaling pathway were up-regulated in Wagyu cattle, in which FGF1, MRAS, RRAS2 and DUSP10 were involved in phosphorylation of ERK1/2. FGF1, MRAS and RRAS2 can indirectly activate ERK1/2 via ERK1/2 signaling pathway, but DUSP10 is JNK/p38-specific phosphatases^47, 48^. Taken together, phosphorylation of ERK1/2 may be higher in subcutaneous adipocytes of Wagyu than Holstein cattle, which may promote adipocyte proliferation. On the basis of these research results about genes and signaling pathways above, we speculated that the subcutaneous adipocyte of Wagyu cattle may show a higher capability of adipogenesis and proliferation than that of Holstein cattle. In conclusion, the fat deposition with significant characteristics in Wagyu cattle is regulated by many transcription factors, genes, and signaling pathways, all of which form a complex regulation network (Fig. 6), but which need to be further verified.

By constructing protein-protein interaction network for differentially expressed genes, we found some key genes: EGR1, FOS, SERPINE1, AGT and MMP2. The study indicates EGR1 can affect adipocyte functions. It can improve insulin resistance of the body by regulating PI3K/Akt and Erk/MAPK signal balance and expression of adipokines tumor-necrosis factor-α (TNF-α) and IL6 in adipose tissue^49^. In our study, 25 genes are enriched in PI3K-Akt signaling pathway. It is possible that EGR1 regulates fat metabolism and deposition in beef cattle by interacting with genes involved in signaling pathway. In addition, SERPINE1 is also important for insulin resistance. The inhibition of it in 3T3-L1 adipocytes can increase the expression of PPARγ, promote adipocyte differentiation, and decrease insulin resistance^50^. Moreover, the study shows that human subcutaneous adipocytes can release MMP2, which activities regulates adipocyte differentiation^51^. Besides, MMP2 can promote fat biosynthesis in murine^52^. With regard to AGT, it is mainly expressed in liver and white fat tissue. After AGT is silenced in 3T3-L1 cell, triglyceride deposition is decreased, and genes associated with fat biosynthesis, fat metabolism, and inflammatory pathway are also down-regulated^53^. In this study, we also identified three subunits of transcription factor activator protein-1 (AP-1): FOS, FOSB, and JUNB, and all of them are up-regulated in subcutaneous adipose tissue of Wagyu cattle. Studies indicate that induction and expression of AP-1 transcription factor plays an important role in adipocyte differentiation, in which ΔFosB, fos-like antigen1 (Fra-1) and fos-like antigen 2 (Fra-2) can interact with CCAAT/enhancer binding proteins-beta (CEBPB), CCAAT/enhancer-binding protein-alpha (CEBPA), and PPARγ, respectively, further inhibiting adipocyte differentiation and fat biosynthesis in mouse^54–56^. The differentially expressed genes discussed above can regulate adipocyte differentiation and disease associated with fat metabolism. This implies that these genes might regulate adipocyte differentiation, fat metabolism and deposition in cattle by interacting with signaling pathway and genes associated with fat metabolism and adipogenesis in beef cattle.

The main physiological function of adipose tissue is to store energy in the form of triglycerides. Meanwhile, adipose tissue functions as an endocrine organ, and can secret many adipokines, which regulate many biological processes including fat metabolism and deposition, glucose metabolism, insulin sensitivity and immunity^57^. Leptin and IL6 are two important adipokines. Leptin is a kind of hormone encoded by LEP gene and can suppress appetite and reduce food intake in mammals, specifically modify adipocyte metabolism to inhibit lipid accumulation and promote lipid mobilization^58, 59^. Previous studies reveal correlation between serum concentration of leptin and fat content, fat thickness and marbling score may vary from cattle breeds or sites. The relationship is positive or not. In addition, Yang, S. H. *et al*. found there is some linear correlation between mRNA level of Leptin gene and size of the adipocytes in crossbred steers (Wagyu × Holstein cattle)^60, 61^. Expression of IL6 gene in subcutaneous adipose tissue is significantly different between W and H (log2FoldChange = 7.16). In previous studies, expression level of IL6 in adipose tissue and circulating concentrations of IL6 positively correlates with obesity and insulin resistance. Besides, IL-6 can promote production of leptin, inhibit activity of lipoprotein lipase, and further may restrain fat deposition of human *in vitro* ^62^. Moreover, Yang *et al*. suggest a model of IL-6-stimulated lipolysis in porcine adipocytes, in which IL-6 chronically activated ERK1/2 pathway, significantly down-regulate the mRNA and protein expression of PPARγ2 as its targeted gene perilipin 1 (PLIN1), finally modify the lipid droplet surface and allow lipases to hydrolyze triacylglycerol^63^. In our study, Wagyu and Holstein cattle show significant difference in fat accumulation and adipocyte sizes of subcutaneous and intramuscular fat. Overall, up-regulated LEP and IL6 gene might play an important role in subcutaneous and intramuscular fat deposition in Wagyu cattle.

## Conclusions

We applied RNA-seq technology and bioinformatics methods to identify the transcriptomic difference of subcutaneous adipose tissues between Wagyu and Holstein cattle and explore the molecular mechanisms of fat deposition. In the study, 662 differentially expressed genes are identified, many of which are closely related to fat metabolism and accumulation. PPAR signaling pathway and fatty acid metabolism with the genes involved have critical roles in adipogenesis and lipometabolism. Moreover, adipocytokines IL6 and Leptin, in addition the genes, EGR1, FOS, SERPINE1, AGT and MMP2 might have great impacts on adipocyte differentiation and lipidosis. This study can provide useful information for understanding molecular mechanism of fat deposition in beef cattle, and breeding beef cattle with high meat quality, preventing and treating disease associated with fat metabolism.

## Materials and Methods

### Ethics statement

All of the procedures involving animals were approved by the animal care and use committee at Institute of Animal Sciences, Chinese Academy of Agricultural Sciences where the experiment was conducted. All of the experiments were performed in accordance with the relevant guidelines and regulations set by the Ministry of Agriculture of the People’s Republic of China.

### Animals and sample preparation

In this study, five Wagyu and five Holstein cattle around 30-month old were obtained from Beijing Huairou Wagyu Technology CO. (Beijing, China) and Langfang Xingcheng meat production company (Hebei, China), respectively. In details, five fattening Wagyu and Holstein steers around 10 month age were chosen first. Then steers of both breeds were fed a diet with increasing concentrate percentage (34.5%~86.5%) from 10 to 20 months of age. With the increasing roughage percentage (13.5~15.5%), the concentrate percentage of the diet were 86.5 to 84.5% from 20 to 30 month of age at the final fattening period. Bodyweight of Wagyu and Holstein cattle were around 715 kg and 905 kg respectively at slaughter. All animals were free access to water and food under natural lighting. Dorsal subcutaneous adipose tissue between 12th and 13th ribs was sampled within 30 min after slaughter, frozen in liquid nitrogen immediately, and then stored in freezer at −80 °C for subsequent RNA extraction and long-term preservation.

### RNA isolations and pooling

Total RNA was extracted from subcutaneous adipose tissues of individual cattle using TRIzol^TM^ Reagent (Invitrogen, USA) in accordance with the manufacturer’s instructions. The quality and quantity of the RNA samples were analyzed by Bioanalyzer 2100 system using an RNA 6000 Nano kit (Agilent technologies, Inc., Santa Clara, USA). The isolated RNA was treated with RNase–free DNase I (Ambion, Inc., Austin, USA) to avoid any potential genomic DNA contamination. Then, equal amounts of RNA samples from five W individuals were pooled, and those from five H individuals were also pooled (n = 5 per pool).

### Library construction and RNA-seq

Approximately 2 μg of RNA from each pooled sample was used to construct RNA-Seq cDNA libraries W (for Wagyu cattle) and H (for Holstein cattle) for sequencing according to the Illumina^®^ TruSeq™ RNA Sample Preparation protocol. In short, the workflow included isolation of poly-adenylated RNA molecules using poly-T oligo-attached magnetic beads, enzymatic RNA fragmentation, cDNA synthesis, ligation of bar-coded adapters, and PCR amplification. Agilent DNA 1000 kit on a Bioanalyzer 2100 (Agilent technologies, Inc., Santa Clara, USA) was applied to check cDNA size and purity of the cDNA libraries. To guarantee high quality of the libraries, ABI stepOnePlus Real-time PCR System was applied to quantify effective concentration of the cDNA libraries (>2 nM). These libraries were sequenced using an Illumina HiSeq^TM^ 2500 platform with 100 paired-end sequencing at the Novogene Bioinformatics Institute (Beijing, China).

### Read filtering and mapping

Fastx_toolkit software (v0.0.14) was used to process raw reads to remove adaptor sequences and low quality reads, and then the clean reads for each sample were aligned to the bovine reference genome Bos_taurus.UMD3.1 (ftp://ftp.ensembl.org/pub/release-84/fasta/Bos_taurus/dna/) with TopHat software (v2.0.12)^64^.

### Analysis of gene expression level and differentially expressed genes

The uniquely mapped read counts of each gene from W and H libraries were counted by HTseq^65^ package (v0.6.1) and normalized using reads per kilo bases per million mapped reads (RPKM) method. Through RPKM normalization, expression levels of different genes from different samples are comparable. If RPKM > 1, the gene is considered to be expressed in cattle.

For this experiment comparing two biological conditions without replicates, the gene read counts were firstly adjusted by the trimmed mean of M-values (TMM) and then identification of differentially expressed genes was performed using DEGseq^66^ R package, which integrates fisher’s exact test and likelihood ratio test to perform differential expression analysis of digital gene expression data following a binomial distribution. Benjamini-Hochberg method was used for multiple hypothesis testing of p_value of genes. For this experiment without biological replicates, |log2FoldChange| ≥ 1(W vs. H) and adjusted p_value ≤ 0.005 were used as threshold, on the basis of which differentially expressed genes were identified.

### Gene Ontology (GO) and pathway enrichment analysis of differentially expressed genes

ClueGO^67^ plugin of Cytoscape was used for enrichment analysis of differentially expressed genes. GO (http://www.geneontology.org/) classifies functions along three aspects including molecular function, biological process, and cellular component. Pathway enrichment analysis can identify the most important metabolic pathway and signal transduction pathways that differentially expressed genes are involved. Kyoto Encyclopedia of Genes and Genome (KEGG)^68^ (http://www.genome.jp/kegg) is a main public database of pathway and a powerful instrument for analysis of metabolism and metabolic network. On the basis of hypergeometric distribution, the GO terms and pathway that differentially expressed genes are significantly enriched in were calculated. Benjamini-Hochberg method was used to correct the obtained p_value. A corrected p_value ≤ 0.05 was considered to represent significant enrichment of genes.

### Identification of alternative splicing events and Single Nucleotide Polymorphisms (SNPs)

ASprofile^69^ (v1.0) was used to analyze the potential alternative splicing events of each sample, which was classified into 12 categories: Alternative 5′ first exon (transcription start site, TSS), Alternative 3′ last exon (transcription terminal site, TTS), Skipped exon (SKIP), Approximate SKIP (XSKIP), Multi-exon SKIP (MSKIP), Approximate MSKIP (XMSKIP), Intron retention(IR), Approximate IR (XIR), Multi-IR (MIR), Approximate MIR (XMIR), Alternative exon ends (AE), and Approximate AE(XAE). SNPs is the major source of genomic variation, and SNP Calling was performed using GATK2^70^ (Genome analysis toolkit) (v3.2) based on Unified Genotyper algorithm.

### Analysis of the protein-protein interaction network

Based on STRING database (http://string-db.org/), we analyzed the protein-protein interaction network for differentially expressed genes, and further investigated the interaction between differentially expressed genes in subcutaneous adipose tissue of Wagyu and Holstein cattle. Cytoscape was used to visualize the protein-protein interaction network for differentially expressed genes, and find the key genes.

### qRT-PCR verification

Real-time quantitative PCR method was applied to validate the expression level of mRNAs. Briefly, approximately 0.5 μg of the same RNA samples was used for reverse transcription using GeneAmp® PCR System 9700 (Applied Biosystems, USA) to synthesize cDNA templates. qRT-PCR analysis was performed in triplicate with a LightCycler^®^ 480 II Real-time PCR Instrument (Roche, Swiss) using QuantiFast^®^ SYBR^®^ Green PCR Kit (Qiagen, Germany) according to the manufacturer’s instructions. The reaction system was consisting of 1 μL cDNA (1:4 dilution), 5 μL of 2× QuantiFast^®^ SYBR^®^ Green PCR Master Mix (Qiagen, Germany), 0.2 μL of forward primer, 0.2 μL of reverse primer, and 3.6 μL of nuclease-free water. The reaction condition included initial denaturation at 95 °C for 5 min, followed by 40 cycles at 95 °C for 10 s, 60 °C for 30 s, and a final stage of dissociation analysis. The primer sequences were designed in the laboratory and synthesized by Generay Biotech (shanghai, China) based on the mRNA sequences obtained from the NCBI database (https://www.ncbi.nlm.nih.gov/). Bovine β-actin gene was used as an internal reference and the 2^−ΔΔCt^ algorithm was applied to calculate the relative expression level of genes between samples.

### Statistical Analyses

All of the data are presented as the means ± SD. When comparisons were made, a Student’s t-test was performed and p < 0.05 was considered statistically significant.

## Electronic supplementary material

Supplementary Information

Table S1

Table S2

Table S3

Table S4

Table S5

Table S6

Table S7
