# Supplementary material for: Global transcriptome analysis identifies differentially expressed genes related to lipid metabolism in Wagyu and Holstein cattle
Source: Sci Rep. 2017 Jul 13;7:5278. doi: 10.1038/s41598-017-05702-5 (PMC5509646; doi:10.1038/s41598-017-05702-5)
Supplement: Supplementary file 1 — Supplementary Information [file 41598_2017_5702_MOESM1_ESM.doc]

Supplementary Information

**Global transcriptome analysis identifies differentially expressed genes related to lipid metabolism in Wagyu and Holstein cattle**

Wanlong Huang, Yuntao Guo, Weihua Du, Xiuxiu Zhang, Ai Li, Xiangyang Miao^*^

Mineral Nutrition Research Division, Institute of Animal Sciences, Chinese Academy of Agricultural Sciences, Beijing, 100193, China

^*^Corresponding author: Xiangyang Miao, Institute of Animal Sciences, Chinese Academy of Agricultural Sciences, Beijing, 100193, China, Tel: 86-10-62895663, Fax: 86-10-62895663 (China), E-mail: miaoxy32@163.com, [mxy32@sohu.com](mailto:mxy32@sohu.com)

**Table S1.** Differently expressed genes between Wagyu and Holstein cattle adipose tissue.

**Table S2.** Gene Ontology analysis of differently expressed genes between Wagyu and Holstein cattle adipose tissue.

**Table S3.** KEGG Pathway analysis of differently expressed genes between Wagyu and Holstein cattle adipose tissue.

**Table S4.** Alternative splicing events in Wagyu and Holstein cattle adipose tissue.

**Table S5.** Distribution of SNPs in Wagyu and Holstein cattle adipose tissue.

**Table S6.** Protein-protein interaction network of differently expressed genes.

**Table S7.** QRT-PCR validation of the differentially expressed genes.
